# Comparison of clinical MRI liver iron content measurements using signal intensity ratios, *R*_2_ and *R*_2_*

**DOI:** 10.1007/s00261-016-0831-7

**Published:** 2016-07-18

**Authors:** Jurgen H. Runge, Erik M. Akkerman, Marian A. Troelstra, Aart J. Nederveen, Ulrich Beuers, Jaap Stoker

**Affiliations:** 1 Department of Radiology, Academic Medical Center, University of Amsterdam, Meibergdreef 9, 1105AZ Amsterdam, The Netherlands; 2 Department of Gastroenterology & Hepatology, Academic Medical Center, University of Amsterdam, Meibergdreef 9, 1105AZ Amsterdam, The Netherlands

**Keywords:** Magnetic resonance imaging, Iron overload, Hemochromatosis, Blood transfusion, Biomarker, Relaxometry

## Abstract

**Purpose:**

To compare three types of MRI liver iron content (LIC) measurement performed in daily clinical routine in a single center over a 6-year period.

**Methods:**

Patients undergoing LIC MRI-scans (1.5T) at our center between January 1, 2008 and December 31, 2013 were retrospectively included. LIC was measured routinely with signal intensity ratio (SIR) and MR-relaxometry (*R*_2_ and *R*_2_*) methods. Three observers placed regions-of-interest. The success rate was the number of correctly acquired scans over the total number of scans. Interobserver agreement was assessed with intraclass correlation coefficients (ICC) and Bland–Altman analysis, correlations between LIC_SIR_, *R*_2_, *R*_2_*, and serum values with Spearman’s rank correlation coefficient. Diagnostic accuracies of LIC_SIR_, *R*_2_ and serum transferrin, transferrin-saturation, and ferritin compared to increased *R*_2_* (≥44 Hz) as indicator of iron overload were assessed using ROC-analysis.

**Results:**

LIC MRI-scans were performed in 114 subjects. SIR, *R*_2_, and *R*_2_* data were successfully acquired in 102/114 (89%), 71/114 (62%), and 112/114 (98%) measurements, with the lowest success rate for *R*_2_. The ICCs of SIR, *R*_2_, and *R*_2_* did not differ at 0.998, 0.997, and 0.999. *R*_2_ and serum ferritin had the highest diagnostic accuracies to detect elevated *R*_2_* as mark of iron overload.

**Conclusions:**

SIR and *R*_2_* are preferable over *R*_2_ in terms of success rates. *R*_2_*’s shorter acquisition time and wide range of measurable LIC values favor *R*_2_* over SIR for MRI-based LIC measurement.

**Electronic supplementary material:**

The online version of this article (doi:10.1007/s00261-016-0831-7) contains supplementary material, which is available to authorized users.

Various diseases are associated with increased liver iron content (LIC), which may induce or contribute to liver damage [1–3]. Serial measurement of LIC during long-term follow-up and treatment is highly desirable, but repeated invasive measurements are not recommended due to risks of complications of serial liver biopsies. Surrogate biochemical markers including serum ferritin and transferrin-saturation are widely used, but are flawed by limited specificity. Thus, accurate non-invasive MRI-based methods of LIC measurement are used in clinical practice for patients (suspected) with increased LIC [4, 5].

Several types of MRI LIC measurement have been described in the literature. Straightforward in–out phase gradient echo (GRE) shows signal loss at the later echo time (TE) but is only qualitative and easily confounded by the presence of hepatic steatosis. Quantitative approaches include (i) signal intensity ratio (SIR) measurement (e.g., the Gandon method) and (ii) MR-relaxometry. The Gandon method (henceforth referred to as “SIR”) utilizes the liver-to-muscle SIR on differently weighted MRI-scans [6]. This method allows easy and free calculation of the LIC_SIR_, by entering ROI values in an online tool [7]. Hence, assuming the acquisition and placement of regions-of-interest (ROIs) are performed correctly, the method is robust to observer influences. A major limitation is its upper limit of detection of 350 µmol/g (equal to 20 mg/g): changes above that threshold cannot be measured.

MR-relaxometry relies on the calculation of tissue relaxation rates (*R*_2_ and *R*_2_*, the inverse of relaxation times *T*_2_ and *T*_2_*), which increase as iron accumulates and are sensitive to changes in LIC values well above the SIR-threshold. One commercialized *R*_2_ approach using single-echo spin-echo (SE) MRI is the FDA-approved St. Pierre method [FerriScan^®^], performed in 10 min in free-breathing [8]. The per-scan analysis price is ~$300, on top of the costs of the MRI-scan itself. Alternative free-of-charge approaches are available for *R*_2_ using free-breathing or respiratory triggered SE-MRI and for *R*_2_* using single breath-hold GRE MRI [9].

Recent developments in MR-relaxometry include multipeak fat corrections and the use of complex instead of magnitude-only data fitting [10], assessment of the effect of fat suppression on *R*_2_* [11] and the comparison of advanced data fit models [12] and analysis approaches [13].

A comparative study of LIC_SIR_, *R*_2_, and *R*_2_* in 94 patients with β-thalassemia reported high correlations [14]. However, success rates, interobserver agreement, and applicability for diseases other than β-thalassemia were not investigated, nor were serum markers assessed. The latter may be useful to screen for elevated LIC (i.e., >36 µmol/g), saving expensive and limited MRI time. We hypothesize that *R*_2_* is preferable over SIR and *R*_2_ in terms of success rate, acquisition time, and range of detection and over serum values in terms of accuracy in detecting elevated LIC.

In our center, the clinical LIC protocol has included SIR, *R*_2_, and *R*_2_* since 2005, with regular weekly clinical referrals since 2008. The SIR measurement is recommended by the national guideline for hemochromatosis [15]. It is supplemented by *R*_2_ and *R*_2_* measurements to fill the gap caused by the SIR method’s hard cut-off at 350 µmol/g. To investigate our hypothesis, we (i) assessed SIR, *R*_2_, and *R*_2_* LIC measurements and their success rates and interobserver agreement; and (ii) compared the diagnostic accuracies of LIC_SIR_, *R*_2_, and surrogate serum markers for correctly predicting elevated LIC based on increased *R*_2_*_._

## Materials and methods

### Ethical

All data used for this study were acquired in clinical setting and were anonymized prior to analysis. Informed consent was waived by the Medical Research Ethics Committee of the AMC Amsterdam.

### Patients

All MRI-based LIC measurements performed between January 1, 2008 and December 31, 2013 were retrospectively included in this study. As additional measurements were added to the protocol in 2014, only measurements up to end 2013 were included. Clinical diagnosis and—when available—serum markers of iron metabolism (total iron, transferrin, transferrin-saturation, ferritin) were collected and subsequently anonymized by a colleague not otherwise involved in this study.

### MRI

MRI-scanning was performed supine, feet first on a 1.5T Avanto MRI-scanner (Siemens AG, Erlangen, Germany) using phased-array coils (body array and spine coil) for localizers and *R*_2_ and *R*_2_* measurements and the body coil for the SIR measurement [6]. Use of the body coil provided an as homogenous B_1_ field as possible, reducing variation in SIR measurements due to variations of flip angles between patients. For *R*_2_* and *R*_2_, the B_1_ variation is eliminated via the data fit. Breath-hold imaging (localizers, SIR and *R*_2_*) was performed in expiration. Three 10-mm slices with a variable slice gap to cover the liver were equally positioned for all three LIC measurements. Especially for the GRE-based SIR and *R*_2_* measurements, careful B_0_ shimming is important to achieve a homogenous B_0_ field, ensuring correct measurements. Shimming was performed with a shim box covering the field-of-view in the feet-head direction and the contours of the abdomen (i.e., excluding the arms) in the left–right and anterior-posterior directions. The SIR measurement according to Gandon et al. requires five (T1, PD, T2, T2+, and T2++) image weightings with specific TR/TE combinations [6]. Table 1 contains an overview of the relevant scan parameters. Of note, the TE interval used for *R*_2_* was shorter (1.41 ms) than the standard in- and out-of-phase interval (2.26 ms).

**Table 1:** MRI parameters

	SIR	*R*_2_	*R*_2_*
Technique	GRE	SE	GRE
TR (ms)	120	3000–4000^a^	300
TE1 (ms)	Variable [7]	6.2	0.99
ΔTE (ms)	n/a	6.2	1.41
Number of echoes	1	16 (multiecho)	12 (multiecho)
FA (°)	Variable [7]	180	20
FOV (mm × mm)	380 × 285	380 × 285	380 × 285
Acquisition matrix	256 × 256	256 × 256	128 × 96^b^
Reconstruction matrix	256 × 192	256 × 192	256 × 192
Parallel imaging	No	GRAPPA	GRAPPA
Acceleration factor	n/a	2	2
Bandwidth (Hz/pixel)	140	465	1963
Slice thickness	10	10	10
Slice gap	Variable^c^	Variable^c^	Variable^c^
Number of slices	3	3	3
Acquisition time	100 s (5 × 20 s)	9–16 min^a^	20 s

^a^Depending on the patient’s respiratory frequency: one TR per respiratory cycle

^b^Zero-padding was used to fit *R*_2_* acquisition in breath-hold time (20 s)

^c^The slice gap was adjusted per patient so as to cover the whole liver with the three slices

### Data analyses

After inclusion all measurements were checked for correct TRs, TEs, and RF coils using DICOM header information as for SIR measurements, specific TR/TE combinations and the use of the body coil are mandatory. Image quality was assessed by a research trainee (JHR, 4 years of experience) and an abdominal radiologist (JS, 20 years of experience) using a 3-point scale (good/adequate/inadequate). The type of artifact(s) was noted. Measurements with incorrect scan parameters or inadequate image quality were classified unsuccessful.

### ROI-placement

**Fig. 1:**
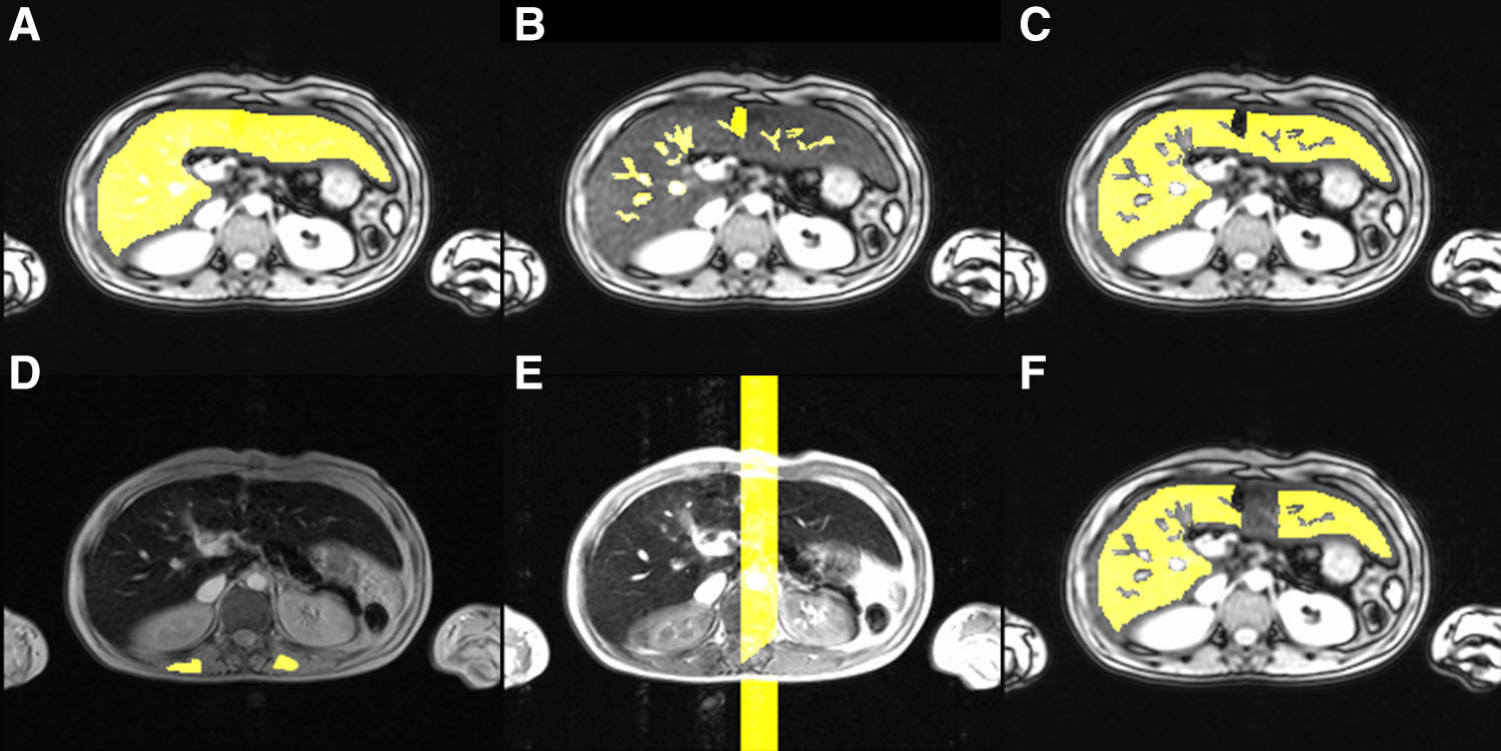
Placement of ROIs**. A**–**F** The placement of ROIs on the data. **A**–**C** How the ROIs for the total liver parenchyma (**A**) and intrahepatic vasculature and/or gall bladder (**B**) are drawn and the result of subtraction in (**C**). **D** The ROI-placement on the paraspinal muscles for SIR calculations. **E**, **F** The placement of a ghosting artifact ROI (**E**) and the final liver parenchyma ROI (**F**) obtained by subtracting (**E**) from (**C**)

SIR, *R*_2_, and *R*_2_* data were processed using custom-made software that allowed ROI-placement, LIC_SIR_ calculation, and *R*_2_ and *R*_2_* data fitting. Three blinded observers (JHR, MAT, and EMA) with four, a half and 9 years of experience, respectively, independently placed regions-of-interest (ROIs) for three slices per scan. First, the liver parenchyma was masked on *R*_2_* source data, excluding a rim near the liver edge (Fig. 1 **A**). Next, non-liver voxels (e.g., vessels, gall bladder) inside the liver contour were masked (Fig. 1 **B**). By subtracting ROI-2 from ROI-1, only liver parenchyma remained (Fig. 1 **C**). Liver ROIs were copied from the *R*_2_* data for SIR analysis, with two additional ROIs in both paraspinal muscles, carefully avoiding areas of signal intensity loss close to the lung (Fig. 1 **D**). This also allowed a check to identify whether patients had moved between *R*_2_* and SIR measurements, in which case new ROIs were placed. Ghosting artifacts caused by aortic blood flow were present in SIR measurements before November 2012 (when saturation slabs were added). Separate ROIs were placed to remove these artifacts from the liver and muscle ROIs (Fig. 1 **E**, **F**). Some reports indicate that susceptibility artifacts may affect *R*_2_* measurements when using a single ROI in liver segments VII or VIII [16]. Due to the limited number of slices, we did not formally assess segmental variations of *R*_2_, *R*_2_*, or LIC_SIR_ in this study.

The respiratory triggering applied for *R*_2_ data acquisition resulted in slight changes in slice positioning so that new ROIs were placed using *R*_2_ source data as described above.

### LIC_SIR_

The calculations published by Gandon et al. were entered into the aforementioned program [7, 17], which automatically chooses the most reliable SIR (i.e., T1, PD, T2, T2+, or T2++) which is converted to LIC_SIR_. The mean LIC_SIR_ of three slices was used and, when one or more values exceeded the 350 µmol/g threshold, the final value was noted as >350 µmol/g. In two subanalyses, the *R*_2_ and *R*_2_* values and the individual SIR ratios in patients with LIC_SIR_ >350 µmol/g were evaluated.

### *R*_2_*

In magnitude images, the noise is distributed in a non-Gaussian manner. This is known as Rician noise [18]. At high signal levels, the non-zero mean has a negligible effect on the average signal, but near the noise level, a noise bias exists which needs to be taken into account when fitting *R*_2_*. We explored three different fit routines: a truncated exponential fit (A) [19, 20], an exponential + constant fit (B) [9, 21], and an exponential + Rician noise (C).

The truncated exponential method A is considered the reference standard, but is time-consuming, where methods B + C do not require further manual input. We compared method B and C with method A as reference using Bland–Altman analysis and *R*_2_* data from a single reader (EMA). Based on this comparison (mean paired difference ($\bar{d}$) was 0.8 Hz for A–C and 33.6 Hz for A–B), we employed method C (Rician noise bias) for the remaining analyses [22, 23].

*R*_2_* calculation was thus performed with a monoexponential model (Eq. 1) with a Rician noise factor. In Eq. 1, *E*_R_ describes the Rice distribution (Online Resource 1), where σ is a noise parameter and $S_{0} \times {\text{e}}^{{ - {R_{2}} ^{*} \times {\text{TE}}}}$ reflects the true magnitude value. Data were averaged inside the ROI before data fitting (average-then-fit).

1 $$ S\left({\text{TE}} \right) = E_{\text{R}} \cdot \left( {S_{0} \cdot e^{{ - {R_{2}}^{*} \cdot {\text{TE}}}} ,\sigma } \right) $$

The effect of intrahepatic fat on *R*_2_* was assessed by applying a biexponential model in a subset (*n* = 10) with definite presence of fat, as identified by the presence of a oscillating signal intensity decay over time. *R*_2_* values with and without correction were compared using Bland–Altman analysis. The ($\bar{d}$) was 0.1 Hz—indicating low overall fat content in this cohort—and deemed negligible compared to the subset mean of 70 Hz. Monoexponentially fitted *R*_2_* values were used for all comparisons.

### *R*_2_

For *R*_2_ calculation an average-then-fit routine was applied using a biexponential model as shown in Eqs. 2 and 3. In Eq. 2, *S*_T_ (TE) is the signal intensity without noise at time TE, *S*_*0*_ is the signal intensity at TE = 0, and *R*_2_ is the relaxation rate. The subscripts *a* and *b* indicate fast and slow relaxation components, respectively. For *R*_2_, Rician noise bias was approximated by the Pythagorean addition of an extra fit parameter, the noise factor ‘*ν*’ in Eq. 3.

2 $$ S_{\text{T}} \left( {\text{TE}} \right) = S_{{{\text{0}},a}} \cdot e^{{ - R_{2,a} \cdot {\text{TE}}}} + S_{{{\text{0}},b}} \cdot e^{{ - R_{2,b} \cdot {\text{TE}}}} $$

3 $$ S\left( {\text{TE}} \right) = \sqrt {S_{\text{T}} \left( {\text{TE}} \right) + \nu^{2} }. $$

In the biexponential model, an iron-dense and an iron-sparse component are assumed, with short and long *R*_2_, respectively. For further comparisons with LIC_SIR_ and *R*_2_*, the bulk *R*_2_ was calculated (Eq. 4) in accordance with the literature [8, 9, 14].

4 $$ R_{2} = \frac{{S_{{{\text{0}},a}} \cdot R_{2,a} + S_{{{\text{0}},b}} \cdot R_{2,b} }}{{S_{{{\text{0}},a}} + S_{{{\text{0}},b}} }} $$

### Comparison with the literature

The relations between the LIC_SIR_, *R*_2_, and *R*_2_* were compared to published regression analysis results based on either biopsy-proven LIC (LIC_BIOPSY)_ [8, 9, 19–21] or LIC_SIR_ [14].

### Statistical analyses

Data are described as number (%) or median (interquartile range, IQR). Results of observers were compared using a Friedman test and Wilcoxon Signed-Rank test as post hoc. Success rates are defined as the number of correctly acquired scans of at least “adequate” quality divided by the total number of measurements. These were compared using a McNemar test. Correlations were assessed with Spearman’s correlation coefficients (*r*_S_), interobserver agreement with two-way random, and absolute intraclass correlation coefficients (ICCs). Both were graded according to Landis et al. [24]. Bland–Altman analysis was performed to compare accuracy between the three MRI methods for a single observer and compare the performance of the three observers [22]. In a separate analysis, the calculated *R*_2_ and *R*_2_* values were converted to ${\text{LIC}}_{R_{2}(\ast)}$ values in μmol/g using the formulas provided by St. Pierre et al. and Garbowski et al. [8, 20] as these were established with image analysis protocols similar to ours.

ROC-analyses were performed for LIC_SIR_, *R*_2_, and serum values with significant correlation with *R*_2_* to establish their diagnostic accuracy to identify increased *R*_2_*, i.e., ≥44 Hz [9]. *R*_2_* was chosen as a reference value as it had the best success rate and shortest acquisition time. The optimal cut-off value for *R*_2_ was found by optimizing the Youden index, while for LIC_SIR_ we used the established cut-off value of >36 µmol/g. *P* values of <0.05 were accepted as statistically significant. Statistical analyses were performed using SPSS Version 22 (IBM Corp, Armonk, NY), MedCalc Statistical Software version 16.2.0 (MedCalc Software bvba, Ostend, Belgium; https://www.medcalc.org; 2016), and GraphPad Prism 5.0 (GraphPad Software, La Jolla, CA).

## Results

### Patients

Between January 1, 2008 and December 31, 2013, a total of 114 patients (M/F: 74/40) underwent 144 MRI-scans for routine LIC measurement. Patient characteristics and clinical indications for LIC measurement are described in Table 2. Thirty patients had multiple measurements. To prevent a repeated measurements effect on correlation assessment between LIC_SIR_, *R*_2_, and *R*_2_*, only the 114 baseline measurements were used. SIR, *R*_2_, and *R*_2_* data were available for 108/114 (95%), 72/114 (63%), and 113/114 (99%) baseline measurements.

**Table 2:** Patient characteristics

	Number (%) or median (IQR)
Patients	114
Male/female	74/40 (65/35%)
Age (years)	44 (28.5–58.1)
Indications	
Sickle cell anemia	21/114 (19%)
MDS^a^/leukemia	19/114 (17%)
Thalassemia	17/114 (15%)
Gaucher’s disease	16/114 (14%)
Hemochromatosis	14/114 (12%)
Hemosiderosis (not specified)	6/114 (5%)
Other	21/114 (18%)

^a^ *MDS* myelodysplastic syndrome

### MRI success rates

Five SIR measurements were classified unsuccessful because a surface coil was used, one due to erroneous TR/TE combinations. Furthermore, image quality was inadequate (respiration artifacts) in a single patient (only *R*_2_ and *R*_2_* acquired). Hence, SIR was successful in 102/114 (89%), *R*_2_ in 71/114 (62%), and *R*_2_* in 112/114 (98%) subjects. The success rate of *R*_2_ was lower than that of SIR and *R*_2_* (*P* < 0.0001, each). Missing datasets were presumed to not have been scanned, with time constraints and respiratory triggering problems as the major cause of the low success rate of the *R*_2_ measurement. For subsequent analyses, only successful baseline measurements were used.

### Interobserver agreement

LIC_SIR_ and *R*_2_ values differed between observer 1 and the other observers (Table 3). However, these differences (median values: 80–85 µmol/g and 33–34 Hz for *R*_2_) would be negligible in clinical practice. This was confirmed by high ICCs for SIR, *R*_2_, and *R*_2_* of 0.998, 0.997, and 0.999, respectively. Bland–Altman analysis between pairs of observers showed a single outlier for SIR, while *R*_2_ and *R*_2_* showed differences up to 5% for higher values, reflecting the uncertainties in the data fit at very high LIC (Online Resource 1).

**Table 3:** MRI interobserver agreement: median (IQR) values

MRI method	Observer 1	Observer 2	Observer 3	*P* value
LIC_SIR_ (µmol/g)	84 (30–205)	80 (25–197)	85 (26–196)	<0.001^a^
*R*_2_ (Hz)	33 (23–48)	34 (24–49)	34 (24–49)	<0.001^a^
*R*_2_* (Hz)	123 (56–321)	126 (55–326)	123 (55–317)	0.092

^a^Post hoc analysis using Wilcoxon Signed-Rank tests showed that LIC_SIR_ and *R*_2_ values of observer 1 differed significantly from either observer 2 or 3 (who did not differ from each other)

### LIC_SIR_, *R*_2_, and *R*_2_*

**Fig. 2:**
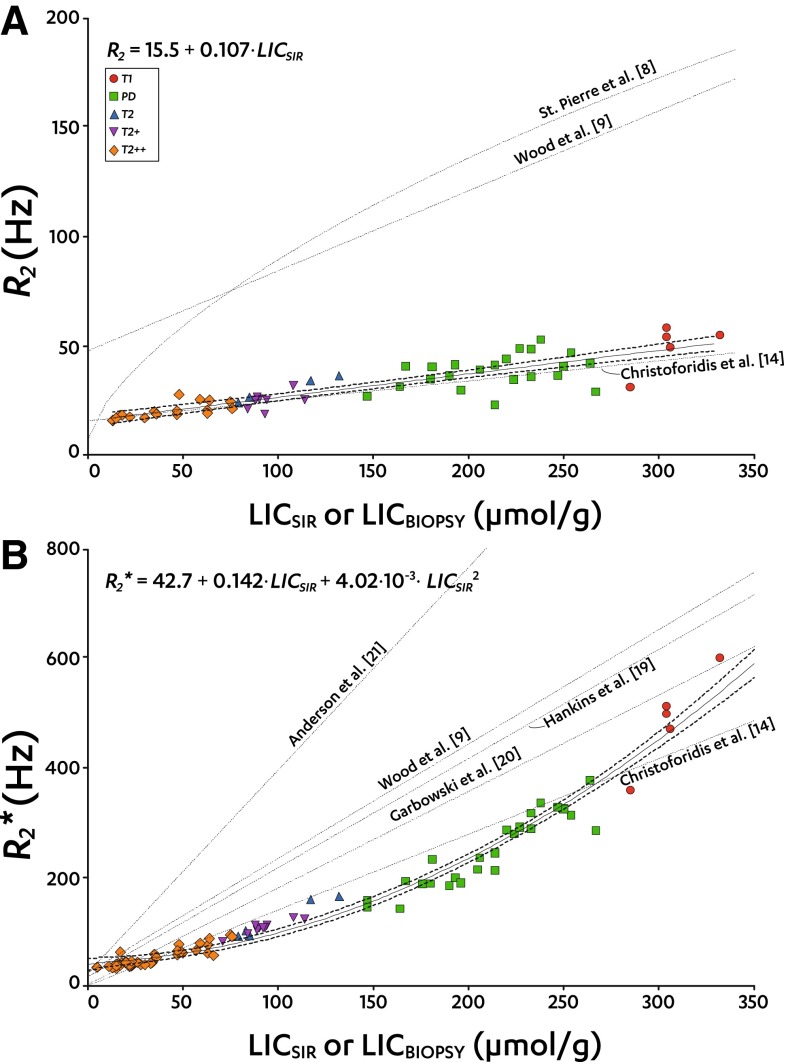
LIC_SIR_ or LIC_BIOPSY_ against *R*_2_ and *R*_2_*. **A**, **B** Scatter plots of LIC_SIR_ against *R*_2_ (**A**, *top*) and *R*_2_* (**B**, *bottom*) for all successful baseline measurements. Data points are grouped by SIR LIC type:  T1;  PD;  T2;  T2+; and  T2++. Regression results (equations given in the figures) are shown by *solid lines*, *with* *dotted* 95% CI bands indicating the goodness of the fit. Additional *dotted regression lines* are based on regression analyses reflecting LIC_BIOPSY_ [8, 9, 19–21] or LIC_SIR_ [14]

Median (IQR) *LIC*_*SIR*_ *, R*_2_, and *R*_2_* (given for observer 1 and LIC_SIR_ <350 µmol/g) were 84 (30–205), 33 (23–48), and 123 (56–321). LIC_SIR_ correlated positively with *R*_2_ and *R*_2_* with *r*_S_ of 0.90 (95% confidence interval (CI) 0.84–0.94, *P* < 0.0001, *n* = 57) and 0.98 (95% CI 0.97–0.99, *P* < 0.0001, *n* = 87), respectively. *R*_2_ correlated positively with *R*_2_*: *r*_S_ of 0.95 (95% CI 0.93–0.97, *P* < 0.0001, *n* = 71). Figure 2 **A**, **B** shows scatter plots of (SIR-based or biopsy-proven) LIC against *R*_2_ and *R*_2_*. Solid lines indicate regression analysis results (95% CI bands as dashed lines). In our patient cohort, *R*_2_ increased linearly with LIC_SIR_ (Eq. 5), while *R*_2_* appeared to have a clear non-linear relationship with LIC_SIR_, well described by a quadratic polynomial (Eq. 6).

5 $$ R_{2} = 15.5 + 0.107 \cdot {\text{LIC}}_{\text{SIR}} $$

6 $$ {R_{2}}^{*} = 42.7 + 0.142 \cdot {\text{LIC}}_{\text{SIR}} + 4.02 \times 10^{ - 3} \cdot {{\text{LIC}}_{\text{SIR}}}^{2} $$

**Fig. 3:**
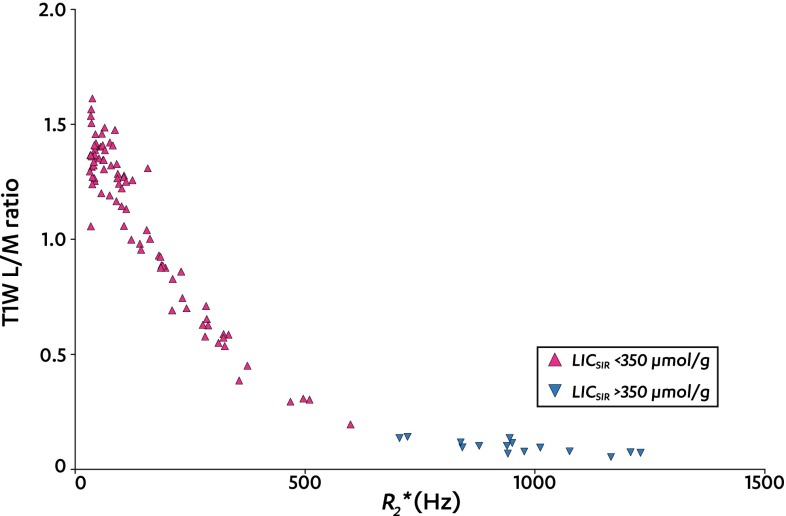
T1W liver-to-muscle SIR against *R*_2_ *. This shows a scatter plot of *R*_2_* values (*x*-axis) against the liver-to-muscle SIR (*y*-axis) of successful baseline T1W SIR measurements. Data are grouped into the following:  LIC_SIR_ <350 and  LIC_SIR_ >350 µmol/g

The LIC_SIR_ upper threshold of 350 µmol/g was reached in 15/102 (15%) measurements. In these measurements, only the T1W SIR correlated with *R*_2_*, with *r*_S_ of −0.72 (95% CI −0.9 to −0.31, *P* = 0.003, *n* = 15). Figure 3 shows the T1 W SIR against *R*_2_*, indicating that for LIC_SIR_ >350 µmol/g, the discriminatory value of the T1W SIR becomes progressively smaller.

### Comparison with the literature

Figure 2 **A**, **B** also shows published regression lines between either LIC_SIR_ or LIC_BIOPSY_ and *R*_2_ (Fig. 2 **A**) and *R*_2_* (Fig. 2 **B**). Contrary to our finding, these lines indicate a linear increase of *R*_2_* as LIC increases, and a non-linear increase of *R*_2_ as LIC increases. To assess whether this is caused by LIC_SIR_ or by *R*_2_ or *R*_2_*, we applied established conversion formulae to convert our *R*_2_ (Eq. 7) and *R*_2_* (Eq. 8) values to LIC values [8, 20]. We then compared these LIC_*R*2*_ and LIC_*R*2_ values to our LIC_SIR_ values.

7 $$ {\text{LIC}}_{{R_{2} }} \, (\upmu {\text{mol/g}}) = 17.91 \cdot \left( {29.75 + \sqrt {\left( {900.7 - 2.283 \cdot R_{2} } \right)} } \right)^{1.424} $$

8 $${\text{LIC}}_{{R_2}^{*}} \, (\upmu {\text{mol/g}}) = \frac{0.029 \cdot {R_{2}}^{{*}^{1.014}}}{5.585\cdot 10^{-2}}$$

These established conversion formulae show a non-linear relation between *R*_2_ and true LIC (Eq. 7) and linear relation between *R*_2_* and true LIC (Eq. 8). Hence, the scatter plot between LIC_*R*2_* and LIC_SIR_ also revealed a quadratic relation, and that between LIC_SIR_ and LIC_*R*2_ a linear one (data not shown).

### Diagnostic accuracies of LIC_SIR_, *R*_2_, and serum values

Serum total iron, transferrin, transferrin-saturation, and ferritin were available for 56, 56, 54, and 96 out of 114 measurements. All four correlated significantly with *R*_2_*, with best correlation for ferritin at *r*_S_ = 0.80 (*P* < 0.0001, *n* = 94).

**Fig. 4:**
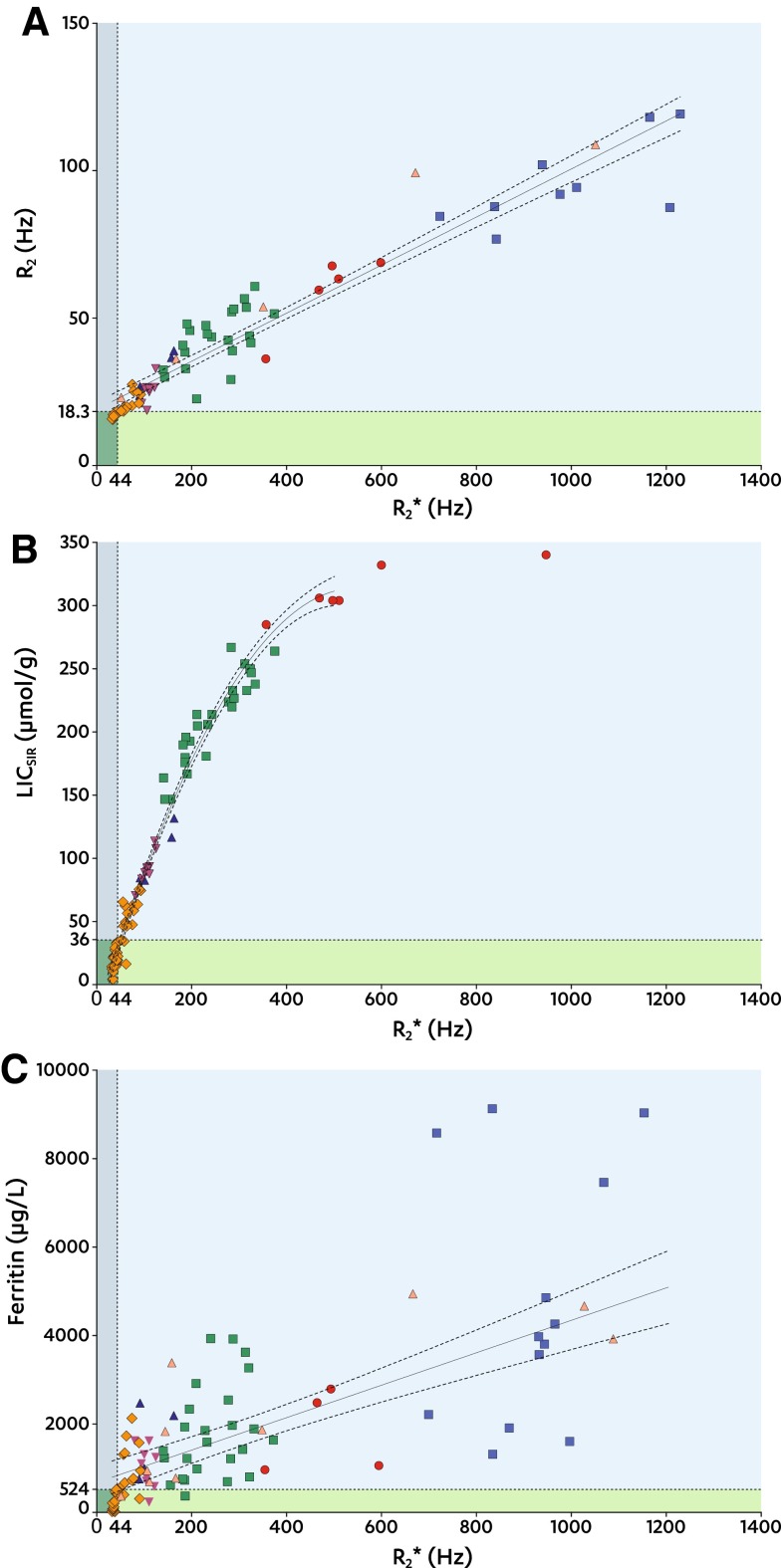
*R*_2_, LIC_SIR_, and ferritin against *R*_2_*. **A**–**C** Scatter plots between *R*_2_* (*x*-axes) and *R*_2_, LIC_SIR_, and serum ferritin (*y*-axes). *Dotted lines* at *x* = 44 and at *y* = 18.3 (**A**), *y* = 36 (**B**), and *y* = 524 (**C**) indicate the thresholds for *R*_2_, LIC_SIR_, and serum ferritin to identify increased *R*_2_* (Table 4). Data points are grouped by SIR LIC type:  T1;  PD;  T2;  T2+;  T2++;  >350; and  no LIC_SIR_ available. Regression results are shown by the *solid lines with dotted* 95% CI bands indicating the goodness of the fit. *Shaded areas* indicate true positive (), true negative (), false positive (), and false negative (), respectively

Increased *R*_2_* (≥44 Hz) was present in 91 subjects. Of the MRI and serum methods, *R*_2_ and ferritin had best diagnostic accuracies to detect increased *R*_2_* (Table 4). Figure 4 **A**–**C** shows true and false positive and negative results of *R*_2_ (Fig. 4 **A**), LIC_SIR_ (Fig. 4 **B**), and ferritin (Fig. 4 **C**) for establishing increased *R*_2_*.

**Table 4:** Diagnostic accuracy values to correctly identify increased *R*_2_* (≥44 Hz)

	*R*_2_	LIC_SIR_	Iron	Transferrin	Transferrin-%	Ferritin
Cases	64/64	75/80	18/41	36/41	20/40	72/80
Cut-off	≥18.3 Hz	≥36 mg/g	≥22.6	≤2.21	≥0.40	≥524
AUROC	1.00 (0.95–1.0)	0.97 (0.91–0.99)	0.66 (0.53–0.79)	0.84 (0.72–0.93)	0.77 (0.64–0.87)	0.98 (0.93–1.0)
Sensitivity	100% (94.4–100%)	93.8% (86.0–97.9%)	43.9% (28.5–60.3%)	87.8% (73.8–95.9%)	50.0% (33.8–66.2%)	90.0% (81.2–95.6%)
Specificity	100% (59.0–100%)	100% (83.9–100%)	100% (76.8–100%)	71.4% (41.9–91.6%)	92.3% (64.0–99.8%)	100% (76.8–100%)
PPV	100% (93.7–100%)	100% (95.2–100%)	100% (82.6–100%)	92.4% (79.8–98.3%)	96.3% (78.3–100%)	100% (94.7–100%)
NPV	100% (77.3–100%)	80.2% (59.7–93.2%)	31.1% (16.7–48.7%)	59.7% (30.3–84.7%)	31.8% (16.4–50.9%)	71.7% (51.0–87.3%)

*AUROC* area under the ROC curve, *PPV* positive predictive value, *NPV* negative predictive value

Values in parentheses reflect the 95% confidence intervals

## Discussion

This study shows that for routine clinical MRI-based LIC measurements SIR and *R*_2_* are more often successful than *R*_2_. Interobserver agreement was near perfect (ICC > 0.9) for all methods. *R*_2_ and *R*_2_* methods provided relaxation rates when the SIR-threshold (>350 µmol/g) was already exceeded. This gives them an advantage over SIR in subjects with transfusional hemosiderosis (at least 55% of our population), when LIC values can easily surpass 350 µmol/g. The combination of high success rate, high interobserver agreement, ability to detect changes in LIC over a wide range of LIC values, and single breath-hold acquisition favors the *R*_2_* method for LIC measurement.

In our study, the relationship between *R*_2_* and LIC_SIR_ was quadratic and remained quadratic when *R*_2_* was expressed as a LIC value using a previously published (biopsy-proven) conversion formula. Other authors report linear relationships. Given the physics of the *R*_2_*–iron relationship, which is basically linear [25], this discrepancy arises either from our *R*_2_* acquisition and analysis or from the reference standard. To rule out the former, we compared three fit routines. The exponential + Rician noise factor fit provided identical results in a fraction of the required time to the established and widely applied but labor-intensive method of manual truncation before exponential fitting.

With respect to reference standard, St. Pierre et al. [8], Wood et al. [9], Hankins et al. [19], Garbowski et al. [20], and Anderson et al. [21] all used biopsy-determined LIC_BIOPSY_ as reference standard, whereas we and Christoforidis et al. [14] used the LIC_SIR_ according to Gandon. Given the similarity of our MRI protocols, it is unsurprising that Christoforidis’ and our data points show considerable overlap. Arguably, their linear relation between LIC_SIR_ and *R*_2_* could also be described by a quadratic polynomial.

Apart from the linear relationship, the other authors report much steeper increase of *R*_2_* as LIC increases [9, 19–21]. Anderson et al.’s very steep increase could be due a long TE1 of 2.2 ms compared to all other studies (range of TE1: 0.8–0.99 ms) that hampers the ability to accurately estimate high *R*_2_* values. The fact that the control values of *R*_2_* in subjects without iron overload in those studies but also in this paper hover around 40 Hz is a further argument that the observed difference in LIC–*R*_2_* does not arise from the *R*_2_* acquisition or analysis but from the reference standard.

Hence, the most likely cause of the deviating quadratic relation between *R*_2_* and estimated LIC is the piecewise sampling of the LIC range with five differently weighted GRE-sequences for LIC_SIR._ This has artificially imposed a quadratic behavior on the actually linear relationship between *R*_2_* and true LIC_BIOPSY_. If one looks at the fundamental GRE signal equation (Eq. 9), where PD is proton density and α is flip angle and applies this to the liver-to-muscle signal intensity ratio, the PD and sin(*α*) terms drop out. By taking the natural logarithm, we find Eqs. 10 and 11. The latter proves that the relationship between *R*_2_* and SIR is logarithmic. Indeed, plotting Fig. 3 with a log-scale for the signal intensity ratio on the *y*-axis linearized the line (data not shown).

9 $$ S\left( {\text{TE}} \right) = \frac{{{\text{PD}} \cdot \sin \left( \alpha \right) \cdot \left( {1 - e^{{ - {\text{TR}}/T_{1} }} } \right)}}{{\left( {1 - \cos \left( \alpha \right) \cdot e^{{ - {\text{TR}}/T_{1} }} } \right)}} \cdot e^{{ - {R_{2}}^{*} \cdot {\text{TE}}}} $$

10 $$ \ln \left( {\frac{{S_{\text{LIVER}} }}{{S_{\text{MUSCLE}} }}} \right) = f\left( {{\text{TR}},\alpha ,T_{1} } \right) + {\text{TE}} \cdot \left( {{R_2}^{*}_{{,\,{\text{LIVER}}}} - {{R_2}^{*}_{,\,\text{MUSCLE}}}} \right) $$

11 $$ {{R_2}^{*}_{,\,\text{LIVER}}} = \frac{{\ln \left( {\frac{{S_{\text{LIVER}} }}{{S_{\text{MUSCLE}} }}} \right) - f\left( {{\text{TR}},\alpha ,T_{1} } \right)}}{\text{TE}} + {{R_2}^{*}_{,\,\text{MUSCLE}}} $$

For *R*_2_, single- and multiecho SE acquisitions are possible: multiecho SE decreases *R*_2_ due to residual signal of stimulated echoes at a given TE. Single-echo SE increases *R*_2_ because long TEs cause increased sensitivity to diffusion, hence increased signal loss at a given TE. Reported single-echo SE *R*_2_ values [8, 9] were concordantly higher for the same estimated LIC compared to multiecho SE results as in this study and in [14]. In terms of *R*_2_ data fitting, we as many others applied a biexponential model and we did not assess non-exponential decay models as for instance proposed by Jensen et al. [26].

The main limitation of our study is the lack of biopsy confirmation. In our center, liver biopsy for iron determination is seldom performed. Both the national, European and American guidelines recommend reluctance in performing biopsy and underline the high sensitivity of MRI [15, 27, 28]. Moreover, differing processing steps to obtain LIC_BIOPSY_ are reported, compromising generalizability. In Gandon’s method, paraffin-embedded liver biopsy specimens are dewaxed using a protocol with a triple xylene wash to remove lipid solids from the sample. This approach was shown to have an elevating effect on the dry weight liver iron calculation compared to processing fresh tissue samples [29]. Another limitation is the fact that we did not perform multipeak fat-correction on complex data [10]. This was not feasible with only magnitude data available. Comparison to other literature is further hampered by the use of different image acquisition and postprocessing protocols which directly influence the calibration curves between the reference standard and the index test. We have opted to compare our findings to calibration curves obtained with similar postprocessing protocols.

ROC-analyses showed that *R*_2_ and ferritin have the highest diagnostic accuracy to identify increased *R*_2_* (≥44 Hz). Both ferritin (≥524 µg/L) and *R*_2_ (≥18.3 Hz) had positive predictive values of 100%, but the wide distribution of ferritin levels for *R*_2_* ≥ 44 Hz indicates that it cannot be used confidently to follow-up treatment nor accurately determine the LIC. In contrast, *R*_2_ shows a different picture with a close distribution around the regression line. In addition, ferritin lacks the spatial information that MRI provides, allowing segmental LIC measurement and follow-up.

*R*_2_ datasets were missing (i.e., not scanned) in 42/114 (37%) subjects. As *R*_2_ is part of our routine scan protocol, this illustrates that the long and artifact-prone *R*_2_ series is skipped first by the radiographer. This makes the *R*_2_ series less suited as first choice for LIC measurement.

Our results favor the use of *R*_2_* measurements for daily clinical practice with the use of an exponential + Rician noise fit method to save time in analysis. The recommendation to (only) use *R*_2_* comes with cautions. It requires careful consideration of scan parameters which should be kept equal for all measurements. Ideally, routine quality control with phantom testing should be performed.

In conclusion, as *R*_2_* can be obtained in a single breath-hold with excellent success rates, high interobserver agreement, and ability to detect changes over a wide range of LIC values and is available from all major vendors without additional per-scan costs, it is our first choice for LIC measurement.

## Electronic supplementary material

Below is the link to the electronic supplementary material.

Supplementary material 1 (PDF 311 kb)

## Abbreviation

LIC: Liver iron content
