# Supplementary material for: Comparison of clinical MRI liver iron content measurements using signal intensity ratios, R2 and R2*
Source: Abdom Radiol (NY). 2016 Jul 18;41(11):2123–31. doi: 10.1007/s00261-016-0831-7 (PMC5059419; doi:10.1007/s00261-016-0831-7)
Supplement: Supplementary file 1 — Supplementary material 1 (PDF 311 kb) [file 261_2016_831_MOESM1_ESM.pdf]

Supplementary Material

Title: Comparison of clinical MRI liver iron content measurements using signal intensity ratios, R_2 and R_2*

Journal name: Abdominal Radiology

Supplementary Material S1

Assessment of three different fit routines for R_2 analysis*

In magnitude images, the noise is distributed in a non-Gaussian manner. This is known as Rician noise (1). At high signal levels the non-zero mean has a negligible effect on the average signal, but near the noise level, a noise bias exists which needs to be taken into account when fitting R_2^*. We explored three different fit routines: a truncated exponential fit (A), an exponential + constant fit (B) and an exponential + Rician noise (C). In A, data points at or below the noise level were deselected manually in a time-consuming process before fitting the remaining points to a single exponential. This method is used most often in literature (2,3). In B, all data points were fit to a single exponential and a constant factor (4,5). The downside of this method is that it introduces a bias to all points, including high signal points and consequently may overestimate the relaxation rate. In C, all points were fit to a single exponential with a Rician noise factor.

The Rician noise factor was modelled by the expectation value (E_R) of the Rice distribution (**eq. 1**), which is subject to two parameters: μ, the 'true' (in this case magnitude) value and σ, a noise parameter. I_0 and I_1 are modified Bessel functions of the first kind.

$$E_R(\mu,\sigma) = \sigma\sqrt{\frac{\pi}{2}}\, e^{-\frac{\mu^2}{4\sigma^2}} \left\{ \left(1+\frac{\mu^2}{2\sigma^2}\right) I_0\left(\frac{\mu^2}{4\sigma^2}\right) + \frac{\mu^2}{2\sigma^2} I_1\left(\frac{\mu^2}{4\sigma^2}\right) \right\} \quad (1)$$

$\mu \gg \sigma$	→	$E_R(\mu,\sigma) = \mu$	**No bias**
$\mu \ll \sigma$	→	$E_R(\mu,\sigma) = \sigma\sqrt{\frac{\pi}{2}}$	**Maximum bias**

To get the fit function for R_2^*, a single exponential is inserted in the place of μ, yielding a function (**eq. 2**) with three parameters: S_0, R_2^* and σ:

$$S(TE) = E_R\left(S_0\, e^{-R_2^*\cdot TE}, \sigma\right) \quad (2)$$

1. Gudbjartsson H, Patz S. The Rician distribution of noisy MRI data. Magn Reson Med 1995;34(6):910-914.
2. Hankins JS, McCarville MB, Loeffler RB, et al. R2* magnetic resonance imaging of the liver in patients with iron overload. Blood 2009;113:4853-4855.
3. Garbowski MW, Carpenter JP, Smith G, et al. Biopsy-based calibration of T2* magnetic resonance for estimation of liver iron concentration and comparison with R2 Ferriscan. J Cardiovasc Magn Reson 2014;16:40.
4. Anderson LJ, Holden S, Davis B, et al. Cardiovascular T2-star (T2*) magnetic resonance for the early diagnosis of myocardial iron overload. Eur Heart J 2001;22:2171-2179.
5. Wood JC, Enriquez C, Ghugre N, et al. MRI R2 and R2* mapping accurately estimates hepatic iron concentration in transfusion-dependent thalassemia and sickle cell disease patients. Blood 2005;106:1460-1465.

Supplementary figure

Figure S1. Bland-Altman plots between pairs of observers for the three methods.

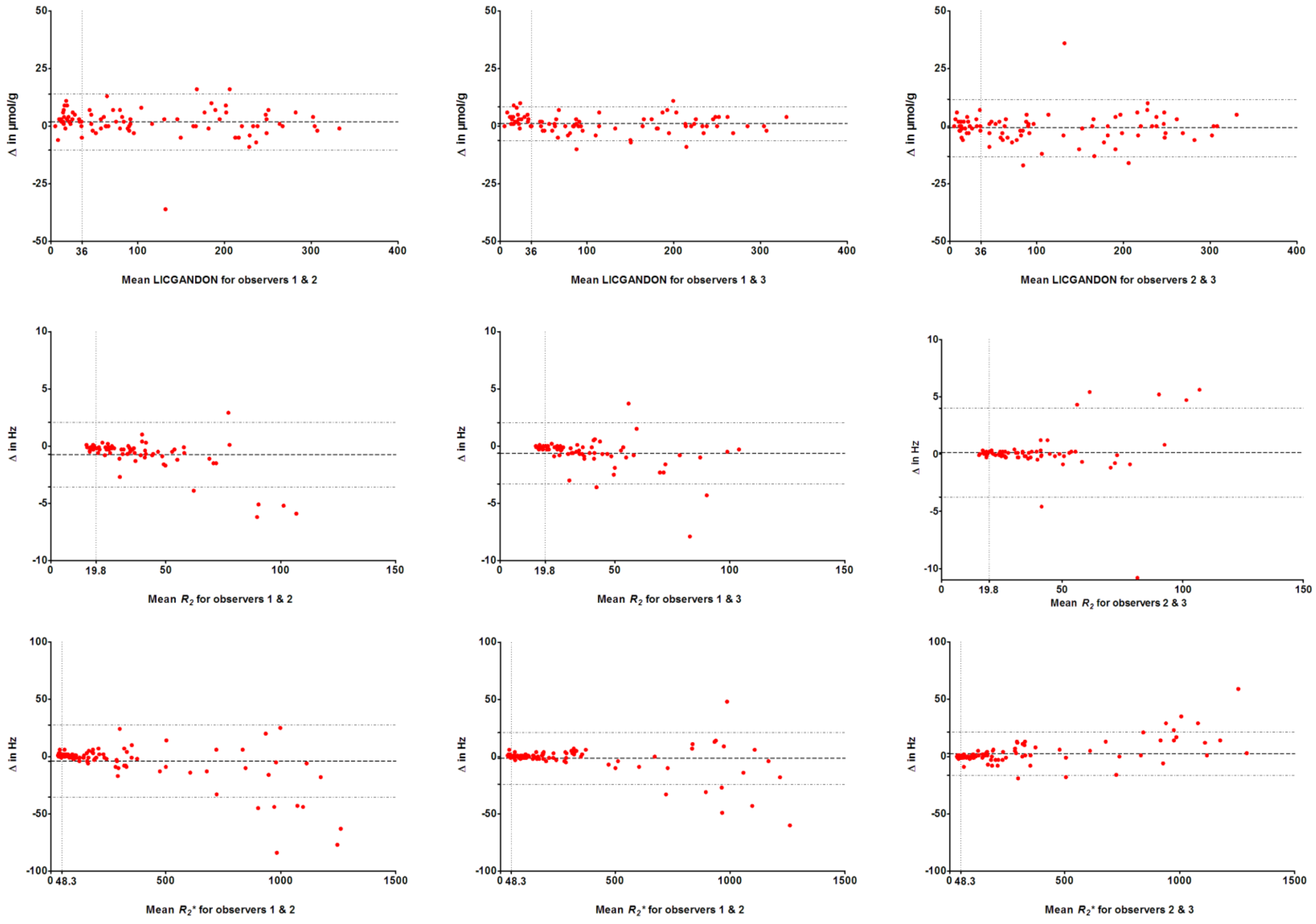

Fig. S1 shows Bland-Altman plots between observer 1 and 2 (*left* column), 1 and 3 (*middle* column) and 2 and 3 (*right* column) for LIC_{GANDON} (*top* row), R_2 (*middle* row) and R_2* (*bottom* row). The dotted vertical lines indicate the thresholds for diagnosing elevated LIC.
